# Macon Medical Society

**Published:** 1885-12

**Authors:** 


### Society Reports.

### MACON MEDICAL SOCIETY.

Reported for THE ATLANTA MEDICAL AND SURGICAL JOURNAL.

STATED MEETING, September 15, 1885.

Dr. J. P. Stevens in the chair.

Dr. R. M. Brown, essayist for the evening, read the following:

### CEREBRAL THROMBOSIS AND EMBOLISM.

These terms imply a condition of the brain which is produced by the accumulation of a clot in the cerebral blood-vessels.

A thrombus, in general, is a clot formed during the life of the patient, either in the cavities of the heart, in the arteries, veins or capillaries. This clot is composed of fibrin, entangling a number of white and red corpuscles of the blood. These may either partially or wholly occlude a vessel.

Physiologists note the formation of these clots in the veins of the mesentery of the frog placed under the microscope. At a given point in the vein there will be seen an accumulation of white blood corpuscles. These are finally seen entangled in a mass of coagulated fibrin, which continues to increase in size until it plugs the vessel. When thrombi are formed they undergo certain changes. If they are of a red color, the first change which they undergo is to become discolorized, so that they resemble thrombi, which were at first white. If the patient continues to live, these thrombi in some instances become organized, being replaced by permanent new connective tissue containing blood-vessels. When this takes place, the blood-vessel in which it occurs becomes permanently obstructed. In other cases, the thrombus, instead of becoming organized, becomes calcified; the fibrin becomes infiltrated with the salts of lime, and then we have the calibre of the vessel occupied by a hard white mass. In other cases, the thrombus degenerates and becomes softened. Part is converted into a thick redish fluid, and the parts which continue solid become brittle, so that portions may be detached and pass into the general circulation.

### CAUSATION.

In order that blood should not coagulate, and should circulate, two things are necessary : First, the blood must be able to be in constant motion ; second, the condition of inner surface of the walls of the blood-vessels must be perfectly normal. A patient may be so enfeebled by disease, or his heart may be working so inefficiently, that the blood is not passed from it into the arteries. The results of the formation of thrombi vary with their particular position. When formed in the heart they may interfere with its action; detached portions may be carried into the circulation, and plug a certain number of arteries or capillaries. When thrombi are formed in the arteries, they commonly produce slow death of the particular part of the body which is supplied by that artery. The part becomes anæmic, gradually dies and degenerates.

Thrombi may give rise to inflammation of the veins; they may produce œdema of the particular part of the body to which the vein belongs, or may produce gangrene, or hypertrophy of the part, or there may be extravasations of blood into it.

When thrombi are formed in the capillaries, they produce the gradual death of the part.

An embolus is a solid body, most frequently a softened thrombus, or rather a portion of one which has passed into the circulation and floats in the blood, and is propelled by the action of the heart into the arteries and capillaries. If it becomes lodged, a thrombus will form about it, and in this way occlusion is produced.

The usual causes of embolism are the presence of thrombi in the veins. If thrombi are situated anywhere in the veins and they degenerate, soften and break down, the parts becoming detached pass into the blood-current and are carried to the right auricle of the heart. The contraction of the right auricle drives them into the right ventricle, and from thence they are forced into the pulmonary artery, and may then pass into the capillaries of the lungs.

If sufficiently small, they pass through the capillaries of the lungs, thence into the pulmonary veins, and to the left auricle of the heart; by the contraction of the left auricle, they are driven into the left ventricle; from thence they pass into the aorta, there to become fixed in some of the small capillaries belonging to the aortic system.

Other causes are vegetation of the valves, tumors projecting into the cavities of the veins; fragments of these may become detached and pass into the circulation, acting as emboli. They must always be derived from a thrombus situated somewhere, but we may be unable to find out the location of this on account of the large number of veins.

### RESULTS OF EMBOLISM.

If an embolus becomes lodged in an artery of some little size, and if this artery is provided with numerous anastomotic branches, the patient feels a shock at the time the embolus becomes lodged. He may feel some pain in the part of the body where the artery is situated. Sometimes the temperature and color of the part is changed. The part sometimes becomes gangrenous.

When an embolus becomes lodged in an artery, not described as having anastomosing branches, the result of the embolism is called infarction, and of this there are two kinds, hemorrhagic and white infarction.

Dr. Moore opened the discussion of the evening with the following paper on

### UTERINE DISPLACEMENTS.

Perhaps no department in medicine offers a wider field for practice, for doing good to suffering humanity, or for garnering professional character, than that of gynæcology, and yet to the average general practitioner hardly any department furnishes so unsatisfactory and uninviting work.

When I first entered the profession, I felt that in the realms of gynæcology I saw fair and inviting fields; that "if the hills of other departments grew dry, I could stray lower down where pleasanter fountains lie." The text-books and teachers of diseases of women spoke in such sanguine language of the curability of the many ills of "dear woman," and the various specula *opened* to imagination's visual organs such intricate and delicate, as well as pleasurable meanderings, it was natural for a young student in medicine to be rather led off in that direction.

As the ardor of our youthful ambition cools off and maturer professional manhood creeps upon us, while we may keep our heads full of theory, and the ways and means for correcting a disordered uterus, yet we shrink from putting into practical execution even our convictions of the proper appliances to bring back an erring uterus to her natural sphere. An old lady once told me that when she was young there were not so many disordered uteruses, and that she never heard of so many womb diseases in her day, and that she believed it was due to the fact that there were so many young doctors a starting out. I wonder if the old lady was not at least half right? Not as applied to the young doctors, but to what might be denominated "meddlesome gynæcology."

It is true that the subject for discussion to night does not go into the broad domain of general gynæcology, yet the same thing may be said of "uterine displacements." I have no question but that many a woman has found an explanation of her troubles in the doctor's diagnosis of some slight uterine displacement which, in fact, either never existed at all, or was so slight as not to give any trouble.

That a great many women, and especially married women, have some deviation from what is described as the normal position of the uterus hardly admits of a doubt, and yet many of them go through life in blissful ignorance of being possessed of an ill-poised uterus. Every man's nose is not just exactly in the same place on the face, or just exactly of the same shape, neither is every woman's uterus just exactly in the same place, nor is it abnormal to find a uterus a little out of the described normal position.

Several years ago I saw, in cousultation with an eminent gynæcologist, a young lady in a state of melancholia which threatened serious insanity, and although there was nothing in the history of the case that would lead to the conclusion that the uterus was to blame for her trouble, yet he insisted upon making an examination, and *thought* that he detected a *slight* anteflexion, with a *little* retroversion, and he saw in this deformity an explanation of all the trouble. I made as careful exploration as I could, but my uneducated touch failed to reveal to me any malposition, though she being a virgin, and having the hymen intact, and the vaginal canal fitting my finger so much more closely than I had ever been accustomed to, may account for my not being able to recognize the displacement. For the sake of my young lady patients, I question the propriety of my educating my sense of touch to this degree of delicacy.

These facts are mentioned for the purpose of laying down what I have for some years felt to be true, viz., that the uterus has charged up to her a great deal of which she is not guilty, and that a great many things are laid to her door which ought not to be.

I come now to inquire what shall be done when we do find this organ out of place, for I am free to say that it does get wrong, and it is our duty to adopt the best means at our hands to correct that wrong.

Now, I understand that the scope of the discussion to-night is to be limited to displacements of the womb that is otherwise healthy, or that is unhealthy only as a *result* of the displacement. In other words, we are to consider displacements that are dependent upon other causes than pregnancy or abnormal growths. It would be impossible in a paper like this to go into anything like a minute investigation of the question of uterine displacements, their individual causes or treatment; hence, I will in a mere general way touch upon the subject.

A great many of the causes which produce *one* displacement are factors in producing *any* or *all* of them; such, for instance, as laborious occupations, habitual constipation, hypertrophy and hyperplasia, loss of tone in the vaginal walls, loss of tone in the uterine ligaments, laxity of abdominal walls, violent coughing or lifting, tumors in the abdomen, ascites, violent muscular eflort, tight and heavy clothing, straining at stool, etc., etc. It is useless to go into a detailed account of the symptoms or the particular management of a displaced uterus. The question which strikes me as the most important in the management of this class of troubles, and the *only* one with which I feel authorized to detain this intelligent society, is that of the use of pessaries. I take the broad position that, as a class, and as a universal remedy, pessaries have done more harm than they ever did good; and while I would not discard them altogether, yet I am strongly of the opinion that the use of the pessary in *any* displacement should be the exception and not the rule. The cure of displacements, especially where they have existed for some time, is not the work of an hour; nor can the cure be easily accomplished while the patient is upon her feet, or is allowed to pursue her usual avocation. Hence, when we take the care of a case of this sort, the first thing to be done is to put your patient upon notice that it may be the work of months, nor can you promise her that your treatment will not interfere with her usual home duties or pleasures.

In prolapsus, whether dependent upon a hyperesthetic condition of the womb or to subjacent surroundings, or a flaccid and relaxed condition of the vaginal walls, or a general want of tonicity in the ligaments and other natural uterine supports, the indications for treatment are the same, viz., reduce the displacement and maintain it in its natural position. Can this be done; if so, how? It is folly to undertake the reduction without placing the patient in the genu-pectoral posture. In a large majority of cases, there will be but little trouble in reducing it in this position. Now the kind of artificial supports to be used to maintain the womb in its place after reduction. In my judgment, no support equals the cotton packing in its universal applicability, but to get the best results, care must be exercised in its use. The cotton packing must be well prepared, thoroughly carded, and everything of an irritating character scrupulously removed; it should then be rolled into small pledgets and properly adjusted, filling the vagina, not tightly, but smoothly and evenly, so as not to press at one point more than another, filling the vaginal canal to the depth of two and one-half or three inches. The cotton may be medicated or not, as indicated by each individual case. If it is desirable to allow the packing to remain several days, it is best to use some sort of disinfectant. I am in the habit of using iodoform ointment, though iodine, carbolic acid, or whatever may be thought best, could be used.

Whenever there is irritation of any character at the os or in the cervical canal, I usually medicate a large pledget of cotton with glycerine and iodized phenol, using a piece large enough to wrap up the entire cervix, and then carefully pack the small dry pledgets around it so as to absorb any oozing of glycerine and secretion. The packing around the os acts upon the principle of pressure by roller bandage, and this, together with a return of the organ to its normal position, soon reduces the inflammation, where it exists, and a consequent subsidence of the concomitant engorgement. By way of parenthesis, I will here remark, that for that condition of the os and cervix which we denominate erosions, where there is no displacement, I have never seen any treatment act so well as the one just described.

The question now very naturally suggests itself as to how long and how constantly should this packing be worn? Dr. Taliaferro, who was one of the first to bring permanently before the profession this practice, at first recommended full and rather *forcible* packing, and *filling* the vagina, and continuing it constantly for a considerable time; but I believe that he has very much modified his plan, both as to the amount of packing and the length of time of wearing it. I satisfied myself six or eight years ago that the forcible, *full* and *continuous* wearing of the packing defeated the very object sought to be accomplished, especially if the prolapse was due to want of tonicity in the vaginal walls, and even when due to other causes, while benefit might be obtained in one direction, there is danger of producing a relaxed and flaccid condition of the vaginal walls, and thereby opening a new source of danger of producing prolapsus. Hence, caution should be exercised in the use of this remedy. I have used the packing with gratifying success when the prolapse was due to want of tonicity in the vaginal and other uterine supports.

When properly used it acts as a stimulant, giving tonicity and contractility to the parts, upon the same principle that the introduction of a large steel bougie would, introduced into the urethra, to restore tone to the genito-urinary functions in the male. But to accomplish this object it should be used not oftener than once or twice a week, and not long enough at a time to overcome the tonic contractility of the parts. This is the plan I try first of all, and am sure that more can be accomplished in this way than by the practice usually pursued. Failing in this, which is seldom, I sometimes resort to other forms of pessaries. I give preference next to Cutter's stem pessary, either passed over the perineum or the symphasis, as will best fill the indication in each individual case, and removing it at night, or when in the recumbent position, to give the parts rest, and replacing again at will. I also supply my patients with a Campbell's uterine repositor, and insist that they keep the womb reduced at any time during the day whenever she feels it to be down, and *especially* at *night*, after retiring for the night, assuming the knee and breast posture. I feel confident that I have cured simple and uncomplicated prolapse of the womb by the use of this little instrument alone.

In the management of retroversions and retroflexions, I have made the cotton-packing,, judiciously and rationally applied, a valuable aid. In these displacements it will be necessary to use great caution in the use of this remedy. After placing the patient in the knee and breast posture, I satisfy myself as to the complete reduction of the organ, even by the rectum if necessary, then place the packing well up behind the uterus, and not fill the entire vagina. In retroflections other measures are to precede the packing. Indeed, I have one general principle in the management of flexions. After having tried a great variety of plans, sounds, spring pessaries, etc., etc., I have simplified my practice in the management of flexions almost entirely to the sponge-tent. To me it has given more general satisfaction than all the other appliances combined; especially is this true of that most troublesome of all displac ments, anteflexion. I introduce as large one as possible at first, and increase as fast as I can until I have dilated the cervical canal large enough to introduce my index finger, and in eight cases out of every ten, when this stage of dilatation has been reached, the flexion is cured, at least sufficiently so for all practical purposes. In the meantime I use the cotton-packing support, as I find it necessary. Should the flexion be too acute to allow the passage of even a small sponge-tent, I commence with a closely rolled cloth-tent, giving it such curve as necessary to pass the flexion; this I follow with a little larger cloth-tent if necessary until I can pass a sponge, and as the sponge swells, and the cervical canal dilates, the flexion is corrected.

The same general principle obtains in the management of any of the flexions and versions, but time will not allow any further ventilation of this question. The other displacements, such as latero-flexions and versions, are rare, and are, upon the same general principles, amenable to the same general management.

Dr. Gewinner fully endorsed Dr. Moore's views, that pessaries had done more harm than good, and reported a case to which he was called in, which patient was suffering from fever, vaginitis, and general pelvic perturbation, with profused leucorrhœa and great nervousness. Upon making digital examination of the vagina, was surprised to find that there was some foreign substance in the vagina. After considerable difficulty, he removed it and found a horse-shoe pessary, which patient had forgotten, and which she had worn two years with great pain and discomfort. The vaginits and other trouble resulting from this pessary was hard to cure, and gave a good deal of trouble. The pessary had been introduced by an itinerant quack.

Dr. Stevens also endorsed the position that much harm had resulted from the use of the pessary, and mentioned the case of a woman who had been treated for a whole year by a gynæcologist in Philadelphia, who had used various pessaries, and the patient came home in a worse fix than when she left. He discarded the pessary treatment, put her upon simple vaginal douché and general tonics, and soon cured her, and she afterwards gave birth to a fine child.

Dr. Ferguson strongly condemned the use of pessaries. From the anatomical construction of the pelvis, and the uterine supports, and its position, can't see how any good can come of the indiscriminate use of all sorts and shapes of pessaries. The only rational pessary, in his judgment, outside the cotton-packing, was Babcock's or McIntosh's stem pessary. Believes that more can be accomplished by constitutional treatment than local.

Dr. R. O. Cotter read a paper on

SOME COMPLICATED CASES OF ULCER OF THE CORNEA.

*Case 1.*—Healthy young man, aged 23. Ulcer at lower periphery of right cornea, caused by purulent (gonorrhœal) ophthalmia. Rapidly progressing through the layers of the cornea, it had reached the posterior, or Descemetian membrane. Patient had fever, considerable intraocular pressure, great pain, etc.

The tension of the eye was very considerable; it threatened rupture of the thin layer of cornea at the ulcer and consequent prolapse of the iris, and perhaps dislocation of the lens and blindness.

As all the principal measures for checking the progress of the ulcer, such as atropia, blisters, cold applications, calomel in purgative doses, morphia, bandages, etc., had been employed without complete success, it was very plain that the intraocular pressure must be relieved. A 4 per cent. solution of cocaine was dropped in the eye and paracentesis of the cornea at the bottom of the ulcer was made almost painlessly. A small iridectomy was also made. The progress of the ulcer was rapidly checked. The other treatment was kept up, and the patient made a rapid recovery. The opacity completely disappeared from the cornea, the small iridectomy left hardly any noticeable deformity, and his vision is now hardly less than normal in this eye.

*Case 2.*—Young woman, aged 19. Very severe purulent ophthalmia in both eyes. By very active treatment, the disease was checked in the right eye, as was also the severer conjunctional symptoms in the left, yet, on the fifth day ulceration began at the lower segment of the cornea of the left eye, and in spite of the closest watching and care, with the utmost cleanliness, use of a two-grain solution of atropia, cold water applications, etc., the ulcer was not sufficiently checked to prevent a very unsightly opacity of the cornea, involving the pupil to some extent, and leaving the patient with vision only in an upward direction in this eye. The other eye was entirely cured. In this case I was of the opinion that paracentesis was not indicated. There was no intraocular pressure and no bulging of the cornea, etc. Purulent ophthalmia, and especially the gonorrhœal form of it, is such a terrible disease that we can afford to congratulate ourselves if we succeed in saving the eye in severe cases, yet in thinking over this case, as in others very similar, it has occurred to my mind that if any successful treatment could have been adopted, it might have been that, after the ulcer first appeared and the great swelling of the conjunctiva and its pressure upon the cornea had been reduced, as it was by the daily or semi-daily application of a ten-grain solution of nitrate of silver (though it was, of course, carefully kept off the cornea)—I say, would a less energetic exhibition of the nitrate of silver solution and a more single use of the two-grain solution of atropia have likely proven more successful? These cases will severely tax our judgment and always require our best skill.

*Case 3.*—Young man, aged 20, was treated for gonorrhœal ophthalmia by Dr. H. J. Williams, of this city. The disease was only in the left eye, the doctor having been so fortunate as to prevent the inoculation of the other. Having about checked the conjunctival inflammation, the Doctor had me to examine the case, as the patient still had a perforating ulcer, one-quarter of an inch in diameter, extending from the edge of the pupil to the periphery of the cornea. There was a prolapse of the iris with inclination towards the periphery. The Doctor had very properly been using a two-grain solution of atropia, and while the inflammatory symptoms were subsiding, the prolapse of the iris was still quite ugly. We decided to leave off the atropia now and use instead a two-grain solution of sulphate of eserine three times daily, and thereby contract the pupil and reduce the prolapse. This, together with a pretty firm bandage, was almost entirely successful—so much so that there remained only a very slight anterior synechia

(adhesion of the iris to the cornea). There still remained, however, a small knuckle of the projecting cornea and iris, and as the constant rubbing of the eye-lid irritated this, a four per cent. solution of cocaine was used, and the hernia carefully picked with a cataract knife, the aqueous humor carefully let off and the deformity was instantly relieved. I will remark just here that if you are not very careful to use a not too stiff speculum, or if you let out the aqueous too suddenly, or allow the patient to squint his eye tightly, you will run a very serious risk of ruining an eye by dislocating the lens.

In this case the eserine was kept up, and although there remained a fistula of the cornea from the puncture, which admitted of the constant flow of the aqueous, and consequent obliteration of the anterior chamber, this fistula was soon cured by the dilatation of the pupil with atropia, as this thickened the iris and caused it to press up against the fistula.

Now, four months after the trouble, the ulcer has healed, and there remains only a very slight anterior synechia and a slightly oblong shape of the pupil. His vision is now 10–20 or very nearly normal. The cicatrix causes some irregularity in the curvature of the cornea, and thus produces some astigmatism, which we hope to relieve after a while by properly adjusted glasses.

*Case 4.*—Negro child, aged 3 years. Decidedly scrofulous; ulcers of both corneas, from phlyctenular ophthalmia, so very common among negroes. These little phlyctenules are very easily checked by careful insufflation of calomel, and perhaps a one or two-grain solution of sulph. atropia dropped in the eye, together with the internal use of iodine in some form, preferably the syr. iodide-iron, together with that sheet-anchor in such conditions, cod liver oil, and also corrected sanitary and dietetic measures. Yet, in this case, as is, unfortunately for the patient, so often in many other cases of eye troubles, the mother purchased a bottle of doctor somebody's eye-water (I don't know what patent eyewash she used) and used it on the child's eyes. These patent eyewashes, I suppose, contain nitrate of silver, or if not, they contain very strong astringents, which if used upon an ulcerated cornea are *highly injurious.* As might have been expected, there was developed, and very quickly, a very extensive ulcer of the cornea, and finally a large prolapse of the iris, and extensive opacity of the cornea, and also a severe iritis.

As is fortunately usual in children, the reparative power of nature is so great that as soon as the child had been gotten thoroughly under the influence of the above-mentioned constitutional remedies, and the corneal and iritic inflammation subdued by the use of a 1 gr. sol. of atropia, a 2 gr. sol. of eserine was used in the eye three times daily, and the prolapse of the iris, which was more formidable than in any of the preceding cases mentioned, was almost entirely relieved. New tissue soon formed over the seat of the ulcer. The opacity is rapidly clearing up, and the child has almost perfect vision already.

*Case 5.*—Old lady, aged 55, in rather feeble health, was affected in right eye with a slow, non-inflammatory ulcer, sickle-shaped, and rapidly extending around the periphery of cornea, and eating deeply into the corneal tissue. Not a great deal of pain, as is usual in these indolent ulcers. No iritis. In this case cold water was studiously avoided, but frequent application of hot water by means of cloths was kept up over the lids (not in the eye), together with a 2 gr. sol. of atropia dropped three times daily in the eye, the hourly administration of egg-nog, milk punch and other good nourishment. The eye was also carefully bandaged to prevent any movement of the lids. I wished to make a paracentisis of the anterior chamber and let off the pressure upon the cornea, but the patient would not by any means consent to the operation. As this class of ulcers are usually caused by a run-down condition of the system, they frequently, I may say usually, attack both eyes, as so happened in this case.

In four or five days the other eye was attacked in precisely the same manner and to as fully a formidable degree as the first eye.

About this time the cornea of the first eye sloughed, and of course this (right) eye became permanently blind. After this result I readily gained the consent of the patient to my making the paracentisis in the left eye, which I did, and by keeping the cut opened by passing in a small probe for two or three days, and thereby letting off the aqueous, and also creating a certain amount of active inflammation, the ulcer gradually healed. The patient has now almost normal vision in this eye.

*Case 6.*—Healthy little girl, aged five years, was referred to me by Dr. W. F. Holt, of this city. As in case number four, the child's mother had used Thompson's eye-water for what she supposed to be a simple "sore eye." The child really had a very small epithelial ulcer of the cornea close to the pupil. Dr. Holt, the family physician, very properly used a one or two-grain solution of atropia, and soon checked the progress of the ulcer, which had in a short time extended in size, involving nearly the whole space between the edge of the pupil and the periphery of cornea. The bad feature of the case was that all the corneal layers, except the Decemetian membrane, had been eaten through, causing quite a formidable bulging, but not yet a perforation of the cornea. While perforation seemed imminent, there was, happily, not a great amount of inflammation and no iritis. Leaving off the atropia now, a two-grain solution of eserine was used three times daily, with the hope of reducing the bulging and breaking up the anterior synechia, and was partly successful. The hernia was also carefully punctured, and the aqueous let off very slowly. A firm bandage was directed also, but owing to the age of the child and the difficulty of controlling her (we having to chloroform her every time we did anything to the eye), it was not kept on. It was found necessary to puncture the protrusion twice afterwards at intervals of four or five days. The last one was made yesterday, the fourteenth instant. For the last four or five days, the plan of alternately dilating and contracting the pupil has worked well—that is, has greatly aided to break up the adhesion. The eye is improving very rapidly now, and I have great reason to hope that the little girl will escape with a slight opacity of the cornea, and perhaps a slightly irregularly shaped pupil. Her vision is normal now.

One word in regard to the use of eserine. It is slightly irritating, and I would warn all against its use, or at least advise its very careful use in those cases where there is a very irritable cornea, or where there is iritis.

Dr. Ferguson reported the case of a patient attacked with intense pain in the left renal region, with red tongue and nausea. Gave fifteen grains calomel followed with acitate of potass.; medicines acted well, and was relieved of headache and nausea. His diagnosis was calculus, and the following day the patient passed a calculus, which the doctor exhibited.

Dr. Stevens reported seeing a gentleman several years ago, at Gowers Spring, who had a tablespoonful of these calculi passed at different times. He claimed that Gowers Spring water cured him.

---

CONSERVATIVE OVARIOTOMY.—Professor Schatz, of Rostock, has described in the *Centralblatt fur Gynakologie*, June 6, a highly interesting case of pregnancy following double ovariotomy performed after a plan recently advocated by Schroder. On February 20, 1880, Dr. Schatz removed from a girl aged 20 a large cystic tumor of the left ovary, including the outer third of the Fallopian tube, and all the ovarian tissue. The right ovary was distinctly enlarged and cystic; it was ligatured by means of three silk threads passed between it and the broad ligament, and cut away in such a manner as to leave a piece of ovarian tissue, hardly two milimetres broad, on the proximal side of the ligature. The right tube remained intact. An abscess formed during recovery in the track of a suture in the abdominal wound. On March 21, when the period was due, severe pain was felt on the right side of the hypogastrium and right thigh, with vomiting and fever. The symptoms recurred on April 8 and May 8. No deposit could be detected in the pelvis. The first "show" appeared on May 9; it lasted three days, and was pale and scanty. It recurred on May 31. In the interval there were attacks of pain in the left groin. On June 11, a swelling of the size of a plum was detected behind and to the left of the uterus, which was strongly anteflexed. On June 28, severe sacral pain set in; it radiated to the left inguinal region, and disappeared at period, which was copious and lasted for six days. On July 15, the uterus was found to be small and retroverted. The catamenia thenceforward appeared regularly till the patient's marriage in April, 1884. She became pregnant in September, and was delivered on May 12 of this year.—*British Medical Journal.*

